# Three levels at which the user's cognition can be represented in artificial intelligence

**DOI:** 10.3389/frai.2022.1092053

**Published:** 2023-01-13

**Authors:** Baptist Liefooghe, Leendert van Maanen

**Affiliations:** Department of Psychology, Utrecht University, Utrecht, Netherlands

**Keywords:** human cognition, user model, explainable AI, cognitive modeling, human behavior

## Abstract

Artificial intelligence (AI) plays an important role in modern society. AI applications are omnipresent and assist many decisions we make in daily life. A common and important feature of such AI applications are user models. These models allow an AI application to adapt to a specific user. Here, we argue that user models in AI can be optimized by modeling these user models more closely to models of human cognition. We identify three levels at which insights from human cognition can be—and have been—integrated in user models. Such integration can be very loose with user models only being inspired by general knowledge of human cognition or very tight with user models implementing specific cognitive processes. Using AI-based applications in the context of education as a case study, we demonstrate that user models that are more deeply rooted in models of cognition offer more valid and more fine-grained adaptations to an individual user. We propose that such user models can also advance the development of explainable AI.

Artificial intelligence (AI) has taken an important place in society and offers support in a variety of domains. Many of these domains require interaction between humans and AI systems, ranging from simple recommender systems to more sophisticated diagnostic tools that are driven by machine learning. Whether this interaction is fruitfully adopted in some cases varies with the degree to which AI adapts to what the user wants, thinks, believes and likes (Baker et al., 2010; Bosse and Hoogendoorn, 2015; Rabinowitz et al., 2018; Bonnefon and Rahwan, 2020; Langley et al., 2022; Nguyen and Gonzalez, 2022). Such adaptation requires the AI system to represent the mental states of the user that are not directly observable and use these states to predict the behavior of the user (Premack and Woodruff, 1978).

A key element endowing AI applications with the ability to adapt to a user is the user model. A user model often consists of a decision-making algorithm that is optimized to provide suitable interventions at the right time given the observable behavior of one or more users (Wahlster and Kobsa, 1989). Following this definition, a user model can be conceptualized as a set of input-output mappings that are learned and can be conceptualized as a subset of a broader context model, which includes all possible situational features that may be relevant, such as time of day, previous interactions, or even seasonal fluctuations (Sporrel et al., 2021; Wang et al., 2021a,b). Hence, human behavior observed by the AI system is not necessarily related to a representation that reflects the user's cognitive state that caused that behavior.

Whereas, a user model that is implemented as a set of input-output mappings may be suitable for some applications, such as recommender systems, the question arises whether this suffices for all domains, or whether there are instances where it is necessary to also represent the cognitive state that underlies user behavior. For instance, it has been argued that the detection of deception by means of AI in the context of airport security is intrinsically flawed because the user model does not appropriately incorporate knowledge of human cognition (Jupe and Keatley, 2020). That is, although AI-algorithms can be trained to detect deception in humans on the basis of facial micro-expressions (Rothwell et al., 2006), psychological research has demonstrated that facial micro-expressions have in fact very low validity in predicting deception (DePaulo et al., 2003). Hence, the relation between behavioral proxy and cognitive construct may not be valid, making the application of micro-expressions to detect deception rather tedious. A similar concern may arise in AI-based recruitment applications that analyze candidates' face expressions and speech demeanor to infer traits such as emotional intelligence and personality (Sethumadhavan and Phisuthikul, 2019; Hmoud, 2021). Even for less circumstantial behavior, such as test performance in an e-learning environment, the question arises whether the response of a participant (e.g., the number of correctly recalled items) reflects some relevant cognitive aptitude (e.g., working-memory capacity) or relates to a spurious factor (e.g., fatigue, distraction, stress,…).

The previous examples thus indicate that in some applications user models need to represent user behavior as well as the mental states underlying that behavior. However, inferring a particular mental state on the basis of a particular observable behavior is often invalid. This is a general problem in cognitive sciences (Borsboom et al., 2004; Poldrack, 2006; De Houwer, 2011; IJzerman et al., 2020) that may contaminate the validity of AI applications as well. The solution to this problem we propose is based on the work of Oberauer and Lewandowsky (2019). These authors distinguish between two types of research in cognitive sciences: discovery-oriented research and theory-testing research. In discovery-oriented research, cognitive models define a search space for the discovery of (behavioral) proxies, but do not entail strong hypotheses by which they can be tested and falsified through the use of these proxies. Theory-testing research relies on cognitive models that do strongly imply such hypotheses and the relation between cognition and behavior is often explicated by formalizing cognitive processes. In the current perspective paper, we argue that implementing user models on the basis of theory-testing research with formal models of human cognition offers a greater insurance that the correct inferences are made by AI applications about a user.

We identify three levels of integration between cognitive theory and user models, ranging from very loose (based on only anecdotal knowledge of cognitive processes) to very tight (implementing the hypothesized cognitive processes in the user model). An overview of these levels is presented in Figure 1. In the next section we elaborate on these levels of integration by considering user models in AI-based applications that assist instruction in educational contexts. We demonstrate that user models that are more strongly integrated with formal models of human cognition, offer greater insurance that AI makes more valid and more fine-grained adaptations to an individual user.

**Figure 1:**
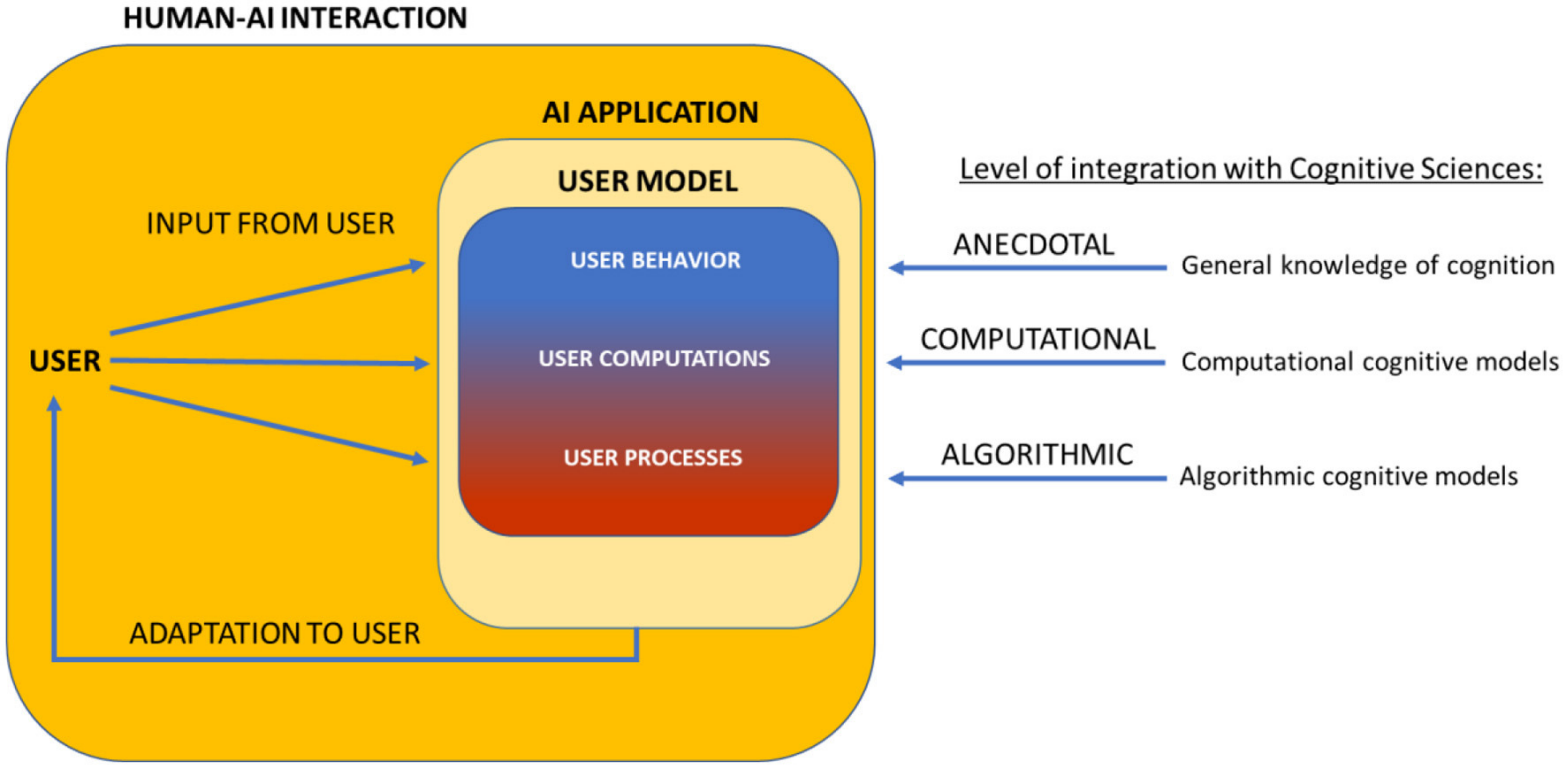
User models in Human-AI interaction can represent the user to different degrees depending on how strongly they are integrated with models from cognitive sciences. At the anecdotal level the user model is inspired from general knowledge of human cognition and does not represent the cognitive state of the user. At the computational level, computations of the user are represented. At the algorithmic level, the user model also incorporates the cognitive processes of the user.

## Three levels of Integration

At the loosest level of integration, the user model is mainly inspired by discovery-oriented research and does not involve any formal specification of human cognition and how it leads to a specific behavior. For instance, it is a generally accepted fact that human short-term memory is limited in capacity (Cowan, 2001). This knowledge could inspire the elaboration of a user model without any explicit specification of the cognitive machinery underlying this capacity limitation. In other words, only general or anecdotal knowledge is used. Hence, we refer to this level as the *anecdotal level* of integration. The second level of integration, the *computational level*, involves user models that consider the computations that are hypothesized to be executed by the users, but ignore the specification of the mental processes giving rise to those computations (Marr, 1982). Building upon our previous example, the user model thus now represents specific short-term memory capacity limits of a user, but does not identify the cognitive processes giving rise to these limitations. The tightest integration can be found at the *algorithmic level*, where a formal specification of mental processes that lead to mental computations is taken into account (Marr, 1982). Within our example, the mental processes leading to a particular short-term memory capacity, such as rehearsal processes, are thus now also formalized in the user model.

We further illustrate these levels in the domain of education in which AI-based applications are used to instruct and learn factual knowledge such as word pairs when learning a foreign language, or a list of all European capitals (Pavlik, 2007; Van Rijn et al., 2009; Sense et al., 2015, 2016, 2018). These applications are inspired by the classic flashcard method [e.g., Pimsleur Language Programs; Leitner (1972) system] in which to-be-learned facts are written out on a deck of cards that are presented one by one. Typically, a question is written on one side of the card, with the answer on the other side. If the learner answers correctly, then the card is put aside, but if the learner answers incorrectly, the card is placed at the bottom of the deck for repetition. When all cards are put aside, the deck is picked up again and the procedure is repeated (Leitner, 1972).

User models of computerized versions of the flashcard method [e.g., SuperMemo, (Wozniak and Gorzelanczyk, 1994)] keep track of users' performance (e.g., errors) so that retention can be improved in three ways. First, by increasing the frequency by which facts are repeated. Consequently, learners display a learning curve or repetition effect, which indicates that performance improves with practice and learning (Ebbinghaus, 1885; Newell and Rosenbloom, 1981; Anderson et al., 1999). Second, by spacing out the presentation of facts evenly in time, which also improves retention [i.e., spacing effect, (Ebbinghaus, 1885; Pavlik and Anderson, 2005)]. Third, by repeatedly testing subjects, which improves recall [i.e., testing effect; (Roediger and Karpicke, 2006a,b)]. These user models have been developed with different levels of integration and vary in the degree to which they can adapt to the user.

At the anecdotal level, the user model registers learning performance and adapts the frequency of repetitions, spacing and number of tests. However, the user model in itself does not include a representation of the mental state of the user or the cognitive processes that are mediating the user's performance. The application thus only adapts to the user's performance on the basis of general principles (repetition effect, spacing effect, and testing effect). Because user performance is directly used as input for this adaptation it is furthermore difficult to control whether this performance reflects the cognitive construct of interest or some uncontrolled mediator (e.g., fatigue) and if the adaption is thus valid.

At the computational level of integration, a representation is made of the user's ability, which underlies the performance of that user, while they execute the task [e.g., (Zhang et al., 2016)]. User models at the computational level can be situated in the Fechnerian tradition of mathematical modeling, which aims to discover functional relationships between observable and metaphysical quantities. To this end, behavioral measures are mapped onto psychological concepts *via* mathematical principles. From this perspective, the user model defines psychological concepts mathematically and behavior is interpreted in light of these concepts. Such an approach is related to cognitive psychometrics [e.g., (Riefer et al., 2002)].

Recent applications in fact learning, which employ user models that are situated at the computational level of integration [e.g., LanguageGarden, (Klinkenberg et al., 2011)] are based on item-response theory [IRT; (Rasch, 1960)]. The idea behind IRT is that the probability that a person has retained a fact (and will answer correctly when tested) is a combination of the difficulty of that fact and the learning ability of the individual. A user model based on IRT simultaneously updates the estimated difficulty of the facts and the estimated ability of the learners by comparing the probability of retaining a fact with the actual outcome on a given test (Klinkenberg et al., 2011; Pelánek, 2016; Pelánek et al., 2017). These estimates are then used to select the next to-be-presented fact. Such a specification ensures that individual learners are presented with facts that are within their reach, but also that facts are repeated in a spaced schedule. This follows because when an individual correctly recalls a fact, both the individual's ability and the item's difficulty are re-estimated, such that the difference between ability and difficulty increases. The adaptations made by the application are thus now based on an explicit representation of the user's cognitive computations that are hypothesized to underlie the observed psychological effects of repetition, spacing and testing.

However, a parameter in a computational model in itself does not guarantee that it reflects a particular feature of cognition and always needs to be validated empirically (Heathcote et al., 2015). Greater validity can be obtained with user models that integrate cognition at the algorithmic level aim to represent the cognitive processes of the user, rather than only the outcome or computations of these processes. To this end, these user models are based on formal models that describe specific cognitive processes [e.g., (van Maanen and Marewski, 2009; Van Maanen et al., 2010)]. An example of an influential formal model that has been used in the domain of fact learning is the ACT-R theory of declarative memory (Anderson and Schooler, 1991a; Pavlik and Anderson, 2005). This theory proposes that memory traces of declarative facts reflect the probability of requiring to recall these facts in the immediate future^1^. This probability or activation is computed on the basis of previous encounters with declarative facts. In particular, the activation is considered to be the highest immediately after a successful recall moment or immediately after a study moment. Following these recall and/or test moments, activation decays with a particular forgetting rate that is specific to the difficulty of the item that is learned and the learning ability of the user. The sum of activation to all encounters of a specific fact determines the probability of needing that fact in the immediate future, as well as a probability of recall of that fact (Anderson and Milson, 1989; Anderson and Schooler, 1991b). This cognitive model of declarative memory has been shown to predict response times and accuracy scores in numerous experiments, including standard memory paradigms (Anderson et al., 1999; Pavlik and Anderson, 2005; Schneider and Anderson, 2012), but also extending to more complex cognitive behavior that involves retrieval of information from memory (Van Rij et al., 2010; Schneider and Anderson, 2011; Banks, 2013).

In the domain of fact learning, the ACT-R theory of declarative memory has been applied in RuggedLearning (Van Rijn et al., 2009; Sense et al., 2015, 2016, 2018). This system uses the activation values of all facts for a particular user to determine which fact has a probability of recall that will drop below a particular threshold in the immediate future. This fact is then selected for the subsequent test. The activation value of that fact is also used to predict a response time of the test. The deviation from the response time is used to calibrate the parameters of the model to best predict the observed recall and response times (Van Rijn et al., 2009). The user model thus now represents assumptions of cognitive processes and adapts the task parameters accordingly. As a result, the user model predicts the effects of repetition, spacing and testing based on the presumed cognitive processes of an individual.

## Discussion

We have illustrated that user models and cognitive models can be integrated at three different levels. Whereas, each level has specific characteristics that can help in the design of a user model, we emphasize that the boundaries between each level are not strict and intermediate levels of integration can be conceived. When considering the different levels of integration in the domain of fact learning, user models designed at the algorithmic and computational level offer greater insurance that the behavior of a user is related to the correct mental states underlying that behavior. The reason for this is that these user models are grounded in formal models of human cognition in which the pathway by which a mental construct leads to a particular behavior is explicated. Accordingly, these models are safer to use in the context of AI applications in which the reverse inference is made, namely from observable behavior to mental construct.

An additional advantage of user models at the computational and algorithmic level is that they offer new avenues for developing explainable AI. Explainable AI refers to AI systems that attempt to provide insight in their decision-making steps to human operators (Gunning et al., 2019; Babic et al., 2021). Not only does explainable AI serve to improve human-AI interaction, but it also helps to make decisions made by AI-algorithms more transparent in society (Ritter et al., 2017), which has recently been identified as one of the main challenges in the future of AI (Schwartz et al., 2022). In recent years it has been advocated that social sciences plays an important role in improving explainable AI. On the one hand, social sciences have offered insights about what constitutes a good explanation (Miller et al., 2017). On the other hand, social sciences and more specifically experimental cognitive psychology has developed research methods that can help unravel the decision processes that are fulfilled by deep neural networks (Taylor and Taylor, 2021a). Here, we argue that explanations offered by an AI system are incorrect when the relation between observable behavior and underlying associated construct is wrong. For instance, if an AI application targets an invalid behavioral proxy, e.g., facial expression as in indicator of threat, then this also invalidates also the explanation provided by that system for the decision it made.

We propose that by using computational or algorithmic user models, AI decision-making can be understood by examining the cognitive models that explicate the reasoning steps taken by the AI system. For example, the aforementioned RuggedLearning application estimates a *rate of forgetting parameter* for each individual user. This parameter informs the decision to adapt the sequence of factual information that needs to be learned. Because the forgetting parameter reflects a relevant cognitive process (that is, memory persistence), it helps to formulate a transparent explanation for the changes made by the AI system. That is, the instruction system provides more learning opportunities, not because general principles of cognition were implemented in the user model (anecdotal level), but because we can identify a parameter value that represents a cognitive process (algorithmic level). Specifically, RuggedLearning provides more learning opportunities when the rate of forgetting parameter is low, *because* the individual has more difficulty retaining the facts.

Of course, we acknowledge that user models not only require the formal representation of cognitive traits such as the user's learning ability. Also more social traits of the user, such as attitudes, likes and dislikes are important. Hence, user models will need to find inspiration in a broad range of (social) cognitive models that formalize personality traits and attitudes [e.g., (Broekens et al., 2013; Moutoussis et al., 2014; Bosse, 2017; Dalege et al., 2018)] in addition to cognitive processes. For instance, a formal theory specifying how particular facial expressions relate to their corresponding mental state would advance the intelligent detection of deception on the basis of facial expressions (Rothwell et al., 2006), validating the decisions made by the AI application (DePaulo et al., 2003) and increasing trust in the system (Ishowo-Oloko et al., 2019). In addition, even if the development of cognitive models leads to a greater scrutiny in the interpretation of behavioral proxies, it remains possible that, over time, a computational or algorithmic model of human cognition proves wrong or incomplete. Consequently, a tight integration with a particular theory of cognitive processing might eventually yield incorrect or suboptimal decisions by the AI system [cf. alternative models in the fact learning domain (Khajah et al., 2014; Lindsey et al., 2014)]. Similarly, algorithmic and even computational models may not be readily available, forcing developers to adopt the anecdotal level until tighter levels of integration become available.

Cognitive theories and models may become invalid over time and are often restricted to a specific domain (e.g., attention, language,…) (Newell, 1973). Accordingly, it could be argued that user models will benefit more by only representing the dynamics of user behavior by means of functional models that formalize the relation between context and behavior without calling upon mediating cognitive processes (Chiesa, 1992). Skinners' conceptualization of teaching machines (Skinner, 1961) offers an early example of how user models can be devised on the basis of the experimental analysis of behavior and formal models are also available in that domain (Mazur, 2006). As pointed out in the Introduction, many user models are functional in nature and useful in a variety of AI applications. However, we believe that such models have difficulties to guarantee that an AI application makes valid inferences about user behavior as they do no consider the cognitive processes underlying that behavior. Furthermore, it has been questioned whether it is computationally possible for AI to make complex inferences about a user, when only observable behavior is available (Armstrong and Mindermann, 2018) and using (formal) models of human cognition may be helpful to mitigate this problem (Hélie and Pizlo, 2022; Langley et al., 2022).

The current perspective can be considered in view of recent developments to endow AI with a Theory of Mind (Premack and Woodruff, 1978) [ToM, e.g., Baker et al., 2010; Cuzzolin et al., 2020; Nguyen and Gonzalez, 2022; for a review see, Langley et al. (2022)]. Whereas a review of these developments is beyond the scope of the present endeavor, we believe that the levels of integration we propose can be helpful when researchers in AI seek inspiration in cognitive (neuro)sciences to develop Machine ToM. Advances in research on ToM are sometimes based on discovery-oriented research (e.g., Wang et al., 2017), which is may be tedious when making inferences about cognition on the basis of behavior (Oberauer and Lewandowsky, 2019). Developing AI models on the basis of such research will result in an anecdotal level of integration between AI and cognitive sciences. As such, researchers in AI may rather use formal of models of human cognition as a basis for their developments [see Nguyen and Gonzalez (2022) for an example]. In general, our framework can thus help in searching for and critically interpreting research in cognitive (neuro)sciences, which has been under heavy debate (Nosek et al., 2015; IJzerman et al., 2020).

To conclude, previous work already highlighted the importance of cognitive psychology (Taylor and Taylor, 2021b) and cognitive neurosciences (Hassabis et al., 2017) in further advancing insights in AI. Typically, these disciplines are considered to be useful in disentangling the so-called black box of artificial cognition by providing research approaches that were developed for the study of human cognition. In the present endeavor we join this position by demonstrating that cognitive models not only help to understand what was engineered, but can also contribute to the engineering itself.

## Data availability statement

The original contributions presented in the study are included in the article/supplementary material, further inquiries can be directed to the corresponding author.

## Author contributions

Both authors listed have made a substantial, direct, and intellectual contribution to the work and approved it for publication.

## Footnotes

^1^The ACT-R theory of declarative memory is based on a rational analysis of memory (Anderson and Milson, 1989; Anderson and Schooler, 1991b; Oaksford and Chater, 1994). Rational analyses of cognition are typically considered computational models, as they relate the computations of a cognitive system to environmental demands, such as the probability that facts are required. The ACT-R theory of declarative retrieval specifies potential algorithms that provide such computations. Thus, a rational analysis of memory not only provides estimates of cognitive computations or outcomes such as IRT, but also determines explicit assumptions about the cognitive processes that give rise to these parameters.
